# Natural Antibodies and Alloreactive T Cells Long after Kidney Transplantation

**DOI:** 10.1155/2021/7005080

**Published:** 2021-09-30

**Authors:** Nicole M. van Besouw, Aleixandra Mendoza Rojas, Sarah B. See, Ronella de Kuiper, Marjolein Dieterich, Dave L. Roelen, Marian C. Clahsen-van Groningen, Dennis A. Hesselink, Emmanuel Zorn, Carla C. Baan

**Affiliations:** ^1^Erasmus MC Transplantation Institute, Department of Internal Medicine-Nephrology & Transplantation and Pathology, University Medical Center Rotterdam, Rotterdam, Netherlands; ^2^Columbia Center for Translational Immunology, Columbia University Medical Irving Center, New York, USA; ^3^Department of Immunohematology and Blood Transfusion, Leiden University Medical Center, Leiden, Netherlands

## Abstract

**Background:**

The relationship between circulating effector memory T and B cells long after transplantation and their susceptibility to immunosuppression are unknown. To investigate the impact of antirejection therapy on T cell-B cell coordinated immune responses, we assessed IFN-*γ*-producing memory cells and natural antibodies (nAbs) that potentially bind to autoantigens on the graft.

**Methods:**

Plasma levels of IgG nAbs to malondialdehyde (MDA) were measured in 145 kidney transplant recipients at 5–7 years after transplantation. In 54 of these patients, the number of donor-reactive IFN-*γ*-producing cells was determined. 35/145 patients experienced rejection, 18 of which occurred within 1 year after transplantation.

**Results:**

The number of donor-reactive IFN-*γ*-producing cells and the levels of nAbs were comparable between rejectors and nonrejectors. The nAbs levels were positively correlated with the number of donor-reactive IFN-*γ*-producing cells (*r*_s_ = 0.39, *p*=0.004). The positive correlation was only observed in rejectors (*r*_s_ = 0.53, *p*=0.003; nonrejectors: *r*_s_ = 0.24, *p*=0.23). Moreover, we observed that intravenous immune globulin treatment affected the level of nAbs and this effect was found in patients who experienced a late ca-ABMR compared to nonrejectors (*p*=0.008).

**Conclusion:**

The positive correlation found between alloreactive T cells and nAbs in rejectors suggests an intricate role for both components of the immune response in the rejection process. Treatment with intravenous immune globulin impacted nAbs.

## 1. Introduction

Donor-specific anti-HLA antibodies (DSA) play a role in antibody-mediated rejection resulting in graft loss [1]. The clinical relevance of non-HLA antibodies, such as natural antibodies (nAbs), is less clear. nAbs are defined by their ability to bind to multiple different ligands including self- and donor antigens [2].

In 1966, nAbs were described as participants in serological recognition of foreign substances [3]. Generally, nAbs are described as preimmune antibodies in the absence of exogenous antigenic stimulation [4]. The main function of nAbs is protection via the innate immune system against bacterial, viral, and fungal infections by epitope recognition. nAbs are also involved in essential functions of the immune system, including B cell function and regulation, clearance of apoptotic debris, allergic suppression, and protection from tumors [4]. Some studies have reported that nAbs play a role in transplantation. nAbs against the angiotensin II type 1 receptor, apoptotic cells, and ARHGDIB antibodies were associated with graft loss [5–8].

Oxidative stress is one of the nonimmunological risk factors contributing to chronic allograft dysfunction in kidney transplant patients [9–11]. Lipid peroxidation is the consequence of oxidative stress resulting in oxidative damage [12]. Malondialdehyde (MDA) is the end product of oxidative stress and can be determined by measuring MDA concentration [13]. This marker of oxidative stress is associated with proinflammatory reactions [14]. After kidney transplantation, MDA was increased in recipients with delayed graft function [11], chronic rejection [9], and risk for cardiovascular mortality [15], while low levels were found in patients with diabetes after transplantation [16]. Increased levels of IgG nAbs reactive to the oxidized antigen of MDA in the first year of kidney transplantation were associated with graft loss [17]. Patients with either DSA or nAbs had a decreased graft survival compared to patients without antibodies [17].

In the current practice, the humoral alloimmune response is the main barrier of graft survival. However, allorecognition by the cellular immune system is the initiator of graft rejection [18]. Therefore, the cross-communication between T cells and B cells is crucial for graft rejection and survival. The number of donor-reactive IFN-*γ*-secreting memory T cells is associated with rejection [19, 20]. Donor-reactive memory cells can induce alloantibody production, and neutralization of IFN-*γ* inhibits alloantibody production [21]. Therefore, we suggest that high numbers of donor-reactive IFN-*γ*-producing cells are associated with high levels of nAbs to MDA.

It is speculated that an adequate control of the T-cell-dependent humoral immune response will improve graft survival. However, the relationship of circulating effector memory T and B cells long after transplantation and their susceptibility to maintenance calcineurin inhibitor (CNI) and anti-rejection therapy are unknown. In this study, we examined the role of nAbs to MDA long after kidney transplantation and the impact of earlier rejection and rejection therapy on nAbs. In addition, nAbs were correlated with donor-reactive memory cells.

## 2. Materials and Methods

### 2.1. Patients

Consecutive patients (*n* = 145) transplanted between January 19, 2010, and April 9, 2013, on CNI therapy and 5–7 years after kidney transplantation (median: 5.68 (5.13–6.39)) provided written informed consent between June 16, 2017, and April 13, 2018. These consecutive patients were included independent of the occurrence of rejection, induction therapy, or anti-HLA antibodies prior to transplantation. The study was conducted in accordance with the Declaration of Helsinki and its amendments. The study was approved by the Medical Ethical Committee of the Erasmus MC (MEC-2016-718 and NL59284.078.16).

Our standard immunosuppressive regimen consists of induction therapy with an anti-IL-2 receptor blocker (basiliximab) plus tacrolimus, mycophenolate mofetil, and prednisolone. Prednisolone is reduced to 5 mg by month 3 after which the drug is tapered further and completely withdrawn at months 4-5. The target tacrolimus dose concentration after 6 months is 5–7 ng/ml, and for cyclosporine A (when tacrolimus is not tolerated, *n* = 4), it is 50–125 ng/ml.

Plasma and peripheral blood mononuclear cells (PBMCs) were isolated from lithium heparin blood at 5–7 years after transplantation and stored at −80 and −196°C, respectively, until use as described previously [22]. Patient characteristics are described in Table 1. Details of rejection and antirejection treatment are summarized in Table 2. Samples of rejectors were taken after rejection and anti‐rejection treatment. After completion of the study, all biopsies for cause were reviewed by a clinical pathologist (MCCG) in a blinded fashion according to the Banff 2015 classification [23].

### 2.2. Determination of Natural Antibodies to MDA

IgG from patients' plasma samples (*n* = 145) were purified and eluted as described previously [2, 17]. IgG nAbs to oxidized lipid epitope MDA were determined by ELISA as described recently [2, 17]. In brief, high-binding 96-well plates (Corning Life Sciences, Kennebunk, ME) were coated with MDA-modified bovine serum albumin (BSA) and postcoated with Tris-buffered saline supplemented with nonfat dry milk. Purified IgG patients' samples and pooled human sera as standard control were added for 2 hours. After incubation with an anti-human IgG antibody (Jackson ImmunoResearch Labs, West Groove, PA), the plates were developed using 3,3′,5,5′-tetramethylbenzidine (Life Technologies, Carlsbad, CA) and the optical density was measured at 450 nm. nAbs were expressed as arbitrary units.

### 2.3. Anti-HLA Antibodies and DSA

The complement-dependent cytotoxicity cross-match was negative before transplantation in all patients.

Plasma samples were screened for the presence of anti-HLA antibodies using the Lifecodes Lifescreen Deluxe (LMX) kit, according to the manufacturer's manual (Immucor Transplant Diagnostics Inc. Stamford, CT, USA). Samples that were considered positive for either HLA class I (HLA-A, HLA-B, or HLA-C) or HLA class II (HLA-DR or HLA-DQ) antibodies were further analyzed with a Luminex Single Antigen assay, using LABScreen HLA class I and class II antigen beads (One Lambda, Canoga Park, GA, USA) [24]. When anti-HLA Abs were present (mean fluorescence intensity >5000), DSA were determined according to the donor HLA mismatches with the recipient.

### 2.4. IFN-*γ* Elispot

The number of IFN-*γ*-producing cells was determined from peripheral blood mononuclear cells (PBMCs). 54 of 145 patients' PBMCs were available in the −196°C storage. Polyvinylidene fluoride (PVDF) plates (Millipore, Darmstadt, Germany) were prewetted with 70% ethanol for 1 minute. After washing the plate, the wells were coated with monoclonal antihuman IFN-*γ* (U-CyTech Biosciences, Utrecht, the Netherlands) overnight at 4°C. After washing the wells with phosphate-buffered saline (PBS), the wells were blocked according to the manufacturer's protocol (U-CyTech Biosciences).

Patients' PBMCs were thawed and rested overnight to prevent spontaneous spot formation. Triplicates of 1 × 10^5^ patients' PBMCs were incubated with 1 × 10^5^ irradiated (40 Gy) PBMCs derived from the donors. Unstimulated patients' PBMCs served as negative control. Stimulation with PHA (5 *μ*g/ml; 2.5 × 10^4^ patients' PBMCs) served as positive control and obtained >50 IFN-*γ*-producing cells per 2.5 × 10^4^ PBMCs. Cells were incubated in the Elispot plate for 24 hours at 37°C, 5% CO_2_, and 95% humidity to allow spot formation. Thereafter, the wells were firmly shaken-out and washed with PBS, and a diluted biotinylated antihuman IFN-*γ* (U-CyTech Biosciences) was added for a period of 2 hours followed by 1-hour incubation with streptavidin-HRP conjugate (U-CyTech Biosciences) and AEC substrate (U-CyTech Biosciences) until distinct spots emerged within 30 minutes. Colour development was stopped by washing extensively with water. When the Elispot plates were dry, spots were counted automatically by using a Bioreader 6000 Elispot-reader (BioSys GmbH, Karben, Germany). In case of response in unstimulated PBMCs, this response was subtracted from the stimulated response.

### 2.5. Statistical Analysis

The Mann–Whitney *U* test was used to analyze differences in level of nAbs between patient groups. Data were presented as median and interquartile range. Correlation analysis between nAbs and the number of IFN-*γ*-producing cells was performed with the Spearman rank test. Differences in sex, age, one or more previous transplants, and percentage of living donations between patients who experienced rejection and those who did not were analyzed by Fisher's exact test. Two-sided *p* values ≤0.05 were considered significant.

## 3. Results

### 3.1. Patients

Thirty-five out of the 145 patients experienced a rejection episode as defined by the Banff classification [23] (*n* = 18 aTCMR (acute T cell-mediated rejection), *n* = 2 aABMR (acute antibody-mediated rejection), *n* = 3 aTCMR + aABMR, *n* = 8 ca-ABMR (chronic active ABMR), *n* = 2 cTCMR (chronic TCMR), and *n* = 2 ca-ABMR + cTCMR). Eighteen rejections occurred within 1 year of transplantation (median: 0.26 year, range: 0.02–0.96), and 17 rejections occurred after one year posttransplantation (3.7 years (1.2–7.0)). No relation with grade of rejection and level of nAbs was found.

Recipient's age, gender, and percentage of living donation were comparable to patients who experienced a rejection episode and those without rejection (Table 1). In the rejection group, a greater number of patients received a previous kidney graft (8/35 vs. 8/110; *p*=0.03) and more patients had anti-HLA antibodies (11/35 vs. 13/110; *p*=0.05) that were present at 5–7 years after transplantation compared to the nonrejection group. There was no difference in the percentage of patients with DSA between the patient groups. All patients received CNI therapy as maintenance therapy, most patients received tacrolimus (*n* = 141), whereas 4 patients received cyclosporine A.

Most patients (72%) with an early rejection period (≤1 year posttransplantation) experienced aTCMR (*n* = 13), 2 patients had aABMR, and 3 patients had aTCMR + aABMR. Therefore, more patients who experienced an early rejection episode were treated with methylprednisolone pulse therapy consisting of 1000 mg intravenously for three consecutive days compared to patients who experienced late (>1 year posttransplantation) rejections (12/18 vs. 3/17; *p*=0.006) (Table 2). Most patients (59%) with a late rejection episode experienced ca-ABMR (*n* = 8) or ca-ABMR + cTCMR (*n* = 2), 2 patients experienced cTCMR, and 5 patients had TCMR. Therefore, more patients with late rejections were treated with methylprednisolone and a single dose of intravenous immunoglobulin (IvIg) (1 g/kg body weight) compared to patients with an early rejection (8/17 vs. 2/18; *p*=0.03). The number of patients treated with antithymocyte globulin (ATG) or alemtuzumab as rejection therapy was comparable between the two patient groups.

### 3.2. No Correlation between nAbs to MDA and Anti-HLA Antibodies

At 5–7 years after transplantation, 24 patients had anti-HLA antibodies, and in 7/24 patients, these antibodies were DSA. In 121 out of 145 patients, anti-HLA antibodies were not detected.

**Figure 1:**
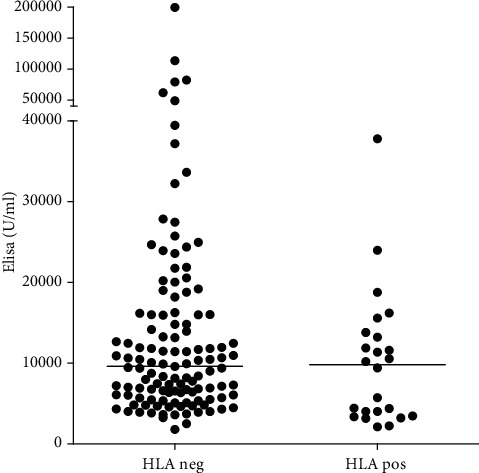
Natural antibodies in patients with and without anti-HLA antibodies.

No difference in nAb levels between patients with and without anti-HLA antibodies nor DSA were found (without anti-HLA: median 9608 U/ml (6060–16018); with anti-HLA 9816 U/ml (3615–13659); DSA positive 4427 U/ml (4024–11890)) (Figure 1).

#### 3.2.1. Correlation between nAbs to MDA and Rejection and Anti-Rejection Treatment

**Figure 2:**
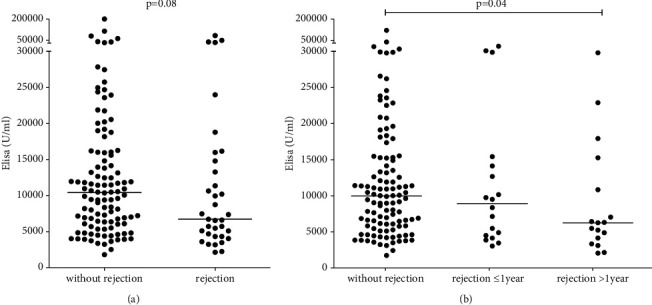
Natural antibodies in patients who have (*n* = 35) or have not (*n* = 110) experienced rejection (a) and those who experienced an early (≤1 year after transplantation, *n* = 18) or late (>1 year after transplantation, *n* = 17) rejection episode (b).

Patients who experienced rejection tended to have lower nAb levels 5–7 years posttransplant than patients without rejection (Figure 2(a); median: 6732 U/ml (4339–12808) vs. 10426 U/ml (6094–16010); *p*=0.08). This difference was mainly present in patients with late rejection episodes (Figure 2(b); late rejection: 6547 U/ml (3908–13666) vs. nonrejection: 10426 U/ml (6094–16010); *p*=0.04).

**Figure 3:**
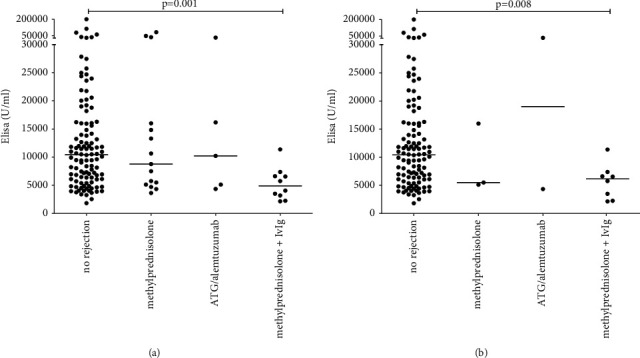
Natural antibodies in patients not treated or treated with anti-rejection therapy (*n* = 110), treated with methylprednisolone alone (*n* = 15), or in combination with antithymocyte globulin (ATG) or alemtuzumab (*n* = 5) or intravenous immunoglobulin (IvIg) (*n* = 10) (a) and only in patients with late rejections (>1 year after transplantation) (b).

Thereafter, we analyzed the impact of previous rejection therapy and the level of nAbs. Patients with previous rejection treatment had different levels of nAbs (Kruskal–Wallis test, *p*=0.03; Figure 3(a)). Patients treated with methylprednisolone followed by IvIg had lower levels of nAbs than patients without rejection episodes (4889 U/ml (2939–6793) vs. 10426 U/ml (6094–16010); *p*=0.001) (Figure 3(a)). This low concentration of nAbs was found in 8 patients with late rejection episodes due to ca-ABMR (6141 U/ml (2551–7177); *p*=0.008) (Figure 3(b)). Because of the lower nAb levels in ca-ABMR samples after antirejection therapy, we found a negative correlation between time between rejection and blood sampling (*r*_s_ = −0.39, *p*=0.02).

#### 3.2.2. Relation between nAbs and IFN-*γ* Elispot

**Figure 4:**
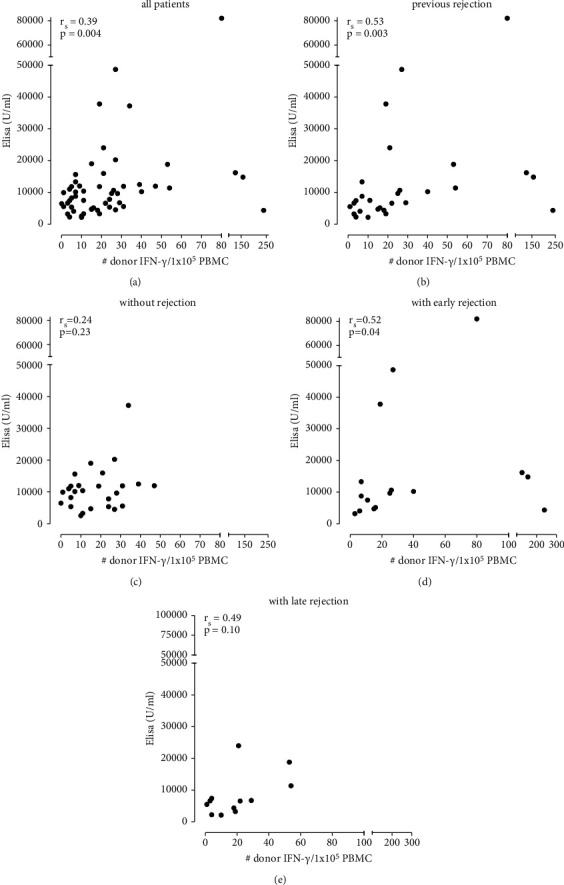
Correlation between natural antibodies and number of donor-reactive IFN-*γ*-producing cells (*n* = 45) (a), in patients with previous rejections (*n* = 28) (b), early rejections (≤1 year, *n* = 16) (d), and late rejection (>1 year, *n* = 12) (e), and in patients without rejections (*n* = 26) (c).

We found a positive correlation 5–7 years posttransplant between the level of nAbs and the number of donor-reactive IFN-*γ*-producing cells (Figure 4(a); *r*_s_ = 0.39, *p*=0.004). The positive correlation was observed in patients who had experienced a rejection episode (Figure 4(b); *r*_s_ = 0.53, *p*=0.003), but not in those without rejection (Figure 4(c); *r*_s_ = 0.24, *p*=0.23). This positive correlation was only found in patients with early rejection episodes (Figure 4(d); *r*_s_ = 0.52, *p*=0.04) and not in those with late rejections (Figure 4(e); *n* = 12, *r*_s_ = 0.50, *p*=0.10). The number of IFN-*γ*-producing cells to HLA mismatched third-party reactivity (*r*_s_ = 0.29, *p*=0.13) and to the positive control (*r*_s_ = 0.20, *p*=0.31), phytohemagglutinin (PHA) stimulation, did not correlate with the level of nAbs.

**Figure 5:**
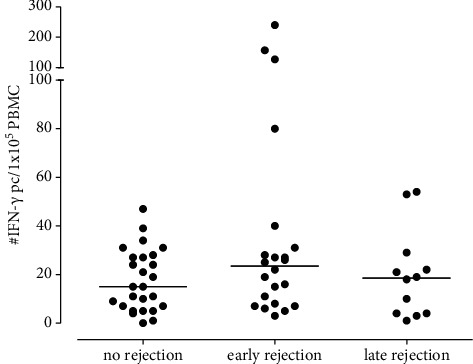
Number of donor-reactive IFN-*γ*-producing cells in patients with early and late rejection and nonrejectors.

No correlation was found between the number of donor-reactive IFN-*γ*-producing cells and patients with early or late rejection and nonrejectors (Figure 5).

The number of donor-reactive IFN-*γ*-producing cells was comparable between the patients with early (24/1 × 10^5^ PBMC (8–33)) and late (19/1 × 10^5^ PBMC (4–27)) rejection episodes and the nonrejectors (15/1 × 10^5^ PBMC (7–27)) (*p*=0.25).

## 4. Discussion

Over the past decades, considerable progress in immunosuppressive therapy to prevent early graft rejection has been achieved. Long-term graft survival rates are largely affected by untreatable ca-ABMR [25, 26]. The presence of DSA could be a risk factor for worse graft function and survival [27]. In recent years, it was shown that nAbs may also have an important function in allograft survival [4–7, 17].

The present paper analyzed the occurrence of nAbs to MDA in patients 5–7 years after kidney transplantation on CNI maintenance therapy and their association with previous rejection episodes and rejection treatment. In addition, the association between nAbs to MDA and the donor-specific cellular response was studied.

It was suggested that nAbs are associated with the formation of anti-HLA antibodies [28]. However, we found no correlation between nAbs and anti-HLA antibodies nor DSA in this cohort of kidney transplant recipients. Apparently, nAbs can develop in the absence of anti-HLA antibodies more than 5 years after transplantation. Only in a few patients (donor-specific), anti-HLA antibodies were found at 5–7 years after transplantation, this might also explain the discrepancy between nAbs and anti-HLA antibodies.

Tissue damage plays an important role in the production of autoantibodies and nAbs, resulting in an enhancement of nAbs during inflammation [29]. Porcheray et al. [30] demonstrated that IgG nAbs against apoptotic cells are present in patients with ABMR. Joosten et al. [31] reported increased production of autoantibodies to the glomerular basement protein agrin in patients with chronic allograft nephropathy. The number of previous rejection episodes was higher in patients with agrin antibodies. See et al. described nAbs at time of rejection and at 1 year after transplantation with a higher grade of rejection [17]. These studies suggest that previous rejections may promote the production of nAbs. The patients in our cohort who experienced a rejection episode more than 1 year after transplantation had significantly lower levels of nAbs to MDA than nonrejectors at 5–7 years after transplantation. Most of these late rejections were ca-ABMR and were treated with methylprednisolone in combination with a preparation of pooled human IgG from plasma from large numbers of healthy blood donors, most commonly referred to as IvIg. The immunomodulatory effects of IvIg are, among other things, dependent on the antibodies present in its contents. IgG autoantibodies and nAbs in IvIg preparations are known to be anti-idiotype and are able to neutralize and immunomodulate autoantibodies and nAbs present in the treated patient [32]. In autoimmune diseases such as acquired hemophilia, vasculitis, systematic lupus erythematosus (SLE), idiopathic thrombocytopenic purpura (ITP), and antiphospholipid syndrome (APS), these antibodies neutralize autoantibodies against factor VIII, antineutrophil cytoplasm, DNA, platelet glycoproteins, and phospholipid autoantibodies, respectively [33]. The anti-idiotype binding to the nAbs of patients treated with IvIg could cause the significant decline in nAb levels of rejectors compared to nonrejectors. The time between ca-ABMR treatment (late rejections) and blood sampling is shorter than the sampling of TCMR (early rejections). It is possible that this difference in time to blood sampling resulted in lower nAbs levels.

The production of nAbs is T cell dependent, mainly CD4^+^ T cells play a role in inducing the level of nAbs [34]. Both nAbs and donor-reactive IFN-*γ*-producing cells are associated with rejection [17, 19, 30], and IFN-*γ* seems to play a role in antibody responses [35]. Additionally, anti-IFN-*γ* mAb treatment inhibited IgG alloantibody responses [21]. If IFN-*γ* has a positive effect on nAbs as it does on IgG alloantibodies, then this could be one of the reasons why we found a positive correlation between nAbs and donor-reactive IFN-*γ*-producing cells, especially in patients with early rejection episodes. Our findings suggest that CNI-based immunosuppressive therapy could not completely deplete effector memory T and B cells. IgG nAbs have the capacity to activate complement, leading to C4d deposition on the surface of target cells, thereby resulting in allograft rejection [6]. Complement activation is able to maintain IFN-*γ* production and sustained endothelial damage of the graft [36]. The positive correlation found between nAbs and the number of IFN-*γ*-producing cells could also be the result of an interaction between nAbs-mediated complement activation and IFN-*γ*-producing T cells.

Our earlier studies [8, 17] described nAbs to MDA at time of rejection and at one year after transplantation. When the nAbs increased at least 50% compared to before transplantation, a relation with rejection was found and worse graft survival until 7 years after transplantation [17]. The present study determined nAbs in a group of patients who had their graft 5–7 years after transplantation and analyzed previous rejection therapy.

The limitations of the study are the low number of patients with DSA at 5–7 years after transplantation and low number of patients with ca-ABMR treated with IvIg. In addition, PBMCs to determine the number of donor-reactive IFN-*γ*-producing cells were only available in 37% of the patients. Therefore, this study should be confirmed in a large prospective study in future.

## 5. Conclusions

To our knowledge, this is the first study to report nAbs to MDA 5–7 years after kidney transplantation. These nAbs were decreased in patients with late rejections, mainly observed in patients treated with IvIg due to ca-ABMR. In addition, a correlation was found between nAbs and donor-reactive IFN-*γ*-producing cells in patients with previous rejection episodes, mainly early rejections within the first year after transplantation. Further studies are necessary to confirm the role of nAbs to MDA in both early and late rejection processes. In summary, both nAbs and donor-reactive IFN-*γ*-producing cells are two components of a complex immune response involved in the rejection process.

## Figures and Tables

**Table 1:** Patient characteristics.

	Without rejection, *n* *=* 110	With rejection, *n* *=* 35	*p* value
*At transplantation*			
Recipient age (median)	55 (44–63)	58 (44–65)	0.26
Recipient gender (% male)	77 (69%)	20 (57%)	0.22
Living donors (%)	85 (77%)	30 (86%)	0.34
Deceased donors (%)	25 (23%)	5 (14%)	0.34
Heart beating deceased donors (%)	9/25	5/5	0.28
Donor age (mean±SD)	49 ± 10	60 ± 14	<0.0001
First transplants (%)	102 (92%)	27 (77%)	0.03

*At 5*–*7 years after transplantation*			
Anti-HLA antibodies (% present)^a^	13 (14%)	11 (31%)	0.05
DSA (% present)^a^	6 (7%)	1 (3%)	1.0
Serum creatinine (µmol/l)	120 (100–141)	152 (127–211)	<0.0001
Tacrolimus trough level (ng/ml)	5.4 (4.7–6.3)	5.8 (4.8–7.6)	0.08
Graft failure at Aug 1, 2021 (%)	4 (4%)	4 (11%)	0.10

^a^Anti-HLA antibodies or DSA determined by Luminex.

**Table 2:** Earlier rejection episodes.

Rejection treatment	Early rejection^a^, *n* = 18	Late rejection^a^, *n* = 17	*p* value
Methylprednisolone	12	3	0.006
Methylprednisolone + rATG^b^	1	0	
Methylprednisolone + alemtuzumab	2	2	
Methylprednisolone + IvIg^c^	2	8	0.03
Patient declined therapy	1	4	0.18

^a^Patients who have experienced early (≤1 year posttransplant) or late (>1 year posttransplant) rejection episodes. ^b^rATG: rabbit antithymocyte globulin. ^c^IvIg: intravenous immunoglobulin.

## Data Availability

The data used to support the findings of the study are included within the article.

## Abbreviations

aABMR: Acute antibody-mediated rejection
aTCMR: Acute T-cell-mediated rejection
ca-ABMR: Chronic active antibody-mediated rejection
CNI: Calcineurin inhibitor
cTCMR: Chronic T-cell-mediated rejection
DSA: Donor-specific anti-HLA antibodies
IFN-*γ*: Interferon gamma
IvIg: Intravenous immunoglobulin
MDA: Malondialdehyde
nAbs: Natural antibodies
PBMCs: Peripheral blood mononuclear cells.
